# Month of Influenza Virus Vaccination Influences Antibody Responses in Children and Adults

**DOI:** 10.3390/vaccines9020068

**Published:** 2021-01-20

**Authors:** Rhiannon R. Penkert, Nehali Patel, Richard J. Webby, Ted M. Ross, Julia L. Hurwitz

**Affiliations:** 1 Department of Infectious Diseases, St. Jude Children’s Research Hospital, Memphis, TN 38105, USA; rhiannon.penkert@stjude.org (R.R.P.); nehali.patel@stjude.org (N.P.); Richard.Webby@STJUDE.ORG (R.J.W.); 2 Department of Microbiology, Immunology and Biochemistry University of Tennessee Health Science Center, Memphis, TN 38163, USA; 3 Center for Vaccines and Immunology, Department of Infectious Diseases, University of Georgia, Athens, GA 30602, USA; tedross@uga.edu

**Keywords:** month, influenza virus, immune response, vaccination, hemagglutination inhibition

## Abstract

The improvement of influenza virus vaccines and the development of a universal product have been long-standing goals in pre-clinical and clinical research. To meet these goals and to understand the strengths and weaknesses of current vaccine strategies, scientists routinely study human responses toward seasonal influenza vaccines. This research is frequently performed with clinical samples taken throughout an influenza season, often without strict attention to the month of inoculation for each study participant. Here, we ask how the timing of vaccination affects outcomes. Results demonstrate significant influences of inoculation month on the immune response. During the progression from fall to winter months, there are changes in host lifestyles and in the frequencies of clinical/sub-clinical viral infections that can significantly alter vaccine immunogenicity. We now recommend routine assessment of inoculation month during clinical studies to inform data interpretation and expedite the development of successful vaccines. This recommendation is pertinent to numerous vaccine development efforts within and outside the influenza virus field.

## 1. Introduction

Influenza virus infections cause serious morbidities and mortalities in children and adults. In 1918, a pandemic killed an estimated 20 to 50 million individuals. Infections also threaten lives in non-pandemic years. During the 2019–2020 influenza season, there were approximately 38 million cases of illness, 400,000 hospitalizations, and more than 20,000 deaths in the United States alone. Licensed influenza virus vaccines confer a degree of protection, but protection is incomplete. There is now a concerted effort to develop better influenza virus vaccines to increase the breadth, durability, and efficacy of the immune response [1].

In an endeavor to improve vaccines and to discover universal products [1,2], scientists define immune correlates by studying responses to candidate and licensed vaccines each year. Often, the hemagglutination inhibition (HAI) assay serves as the gold-standard assay for influenza studies, and variables such as age and sex are evaluated as confounding factors.

Rarely is the month of vaccination considered when vaccine data are evaluated. Therefore we examined HAI titers from two different clinical studies involving (i) children who were vaccinated in 2016–2017 and (ii) adolescents and adults who were vaccinated in 2017–2018. Results from both studies showed that the month of vaccination significantly influenced influenza virus-specific immune responses. Data are pertinent to all vaccine development efforts, both within and outside the influenza virus field.

## 2. Materials and Methods

### 2.1. 2016–2017 Study

A study was performed during the 2016–2017 influenza season at St. Jude Children’s Research Hospital in Memphis, TN (clinical trials.gov #NCT02649192) among 22 healthy children between the ages of two and eight years. These children were participating as controls in a vitamin supplementation study. They received a placebo gummy at the time of vaccination [3]. Vaccines were Fluzone Quadrivalent (Sanofi Pasteur) for children <3 years of age and Fluarix Quadrivalent (GlaxoSmithKline) for children of ≥3 years of age, administered intramuscularly (IM). Serum samples were taken prior to vaccination and 28 days post-vaccination. The recommended composition of the 2016–2017 vaccine was A/California/7/2009 H1N1 pdm09-like virus, A/Hong Kong/4801/2014 H3N2-like virus, B/Brisbane/60/2008-like virus (B/Victoria lineage), and B/Phuket/3073/2013-like virus (B/Yamagata lineage). This study was reviewed and approved by the Institutional Review Board of St. Jude Children’s Research Hospital.

### 2.2. 2017–2018 Study

A study was performed during the 2017-2018 influenza season among 271 participants between the ages of 12 and 83 years. The recommended composition of the vaccine in 2017–2018 was A/Michigan/45/2015 H1N1pdm09-like virus, A/Hong Kong/4801/2014 H3N2-like virus, B/Brisbane/60/2008-like virus (B/Victoria lineage) and B/Phuket/3073/2013-like virus (B/Yamagata lineage). The vaccine was Fluzone, administered IM. Most participants (*n* = 255, 94%) received the standard-dose, quadrivalent vaccine. Among participants >65 years old (*n* = 34), sixteen received the high-dose vaccine (trivalent, lacking the B/Yamagata lineage vaccine component). Serum samples were taken prior to vaccination and 21 days later. This study was reviewed and approved by the Institutional Review Board of the University of Georgia.

### 2.3. HAI Assays

The HAI assay was conducted for the 2016–2017 study with viruses A/California/7/2009 H1N1, A/Switzerland/9715293/2013 H3N2, B/Phuket/3073/2013, and B/Brisbane/60/2008 using turkey RBCs. The HAI assay was conducted for the 2017–2018 study with viruses A/Michigan/45/2015 H1N1, A/Hong Kong/4801/2014 H3N2, B/Phuket/3073/2013, and B/Brisbane/60/2008 using turkey or guinea pig RBCs. The changes between pre-vaccination and day 28 HAI titers (for the 2016–2017 study) or pre-vaccination and day 21 HAI titers (for the 2017–2018 study) were determined. Viruses are termed H1N1, H3N2, Phuket, and Brisbane for simplicity in further text.

### 2.4. Statistical Analyses

GraphPad-Prism software was used for statistical analyses. Medians and interquartile ranges were determined. Rank-based Kruskal–Wallis with Dunn’s multiple comparisons and Mann–Whitney tests were performed to evaluate differences in HAIs between months.

## 3. Results

### 3.1. 2016–2017 Vaccine Study

**Figure 1:**
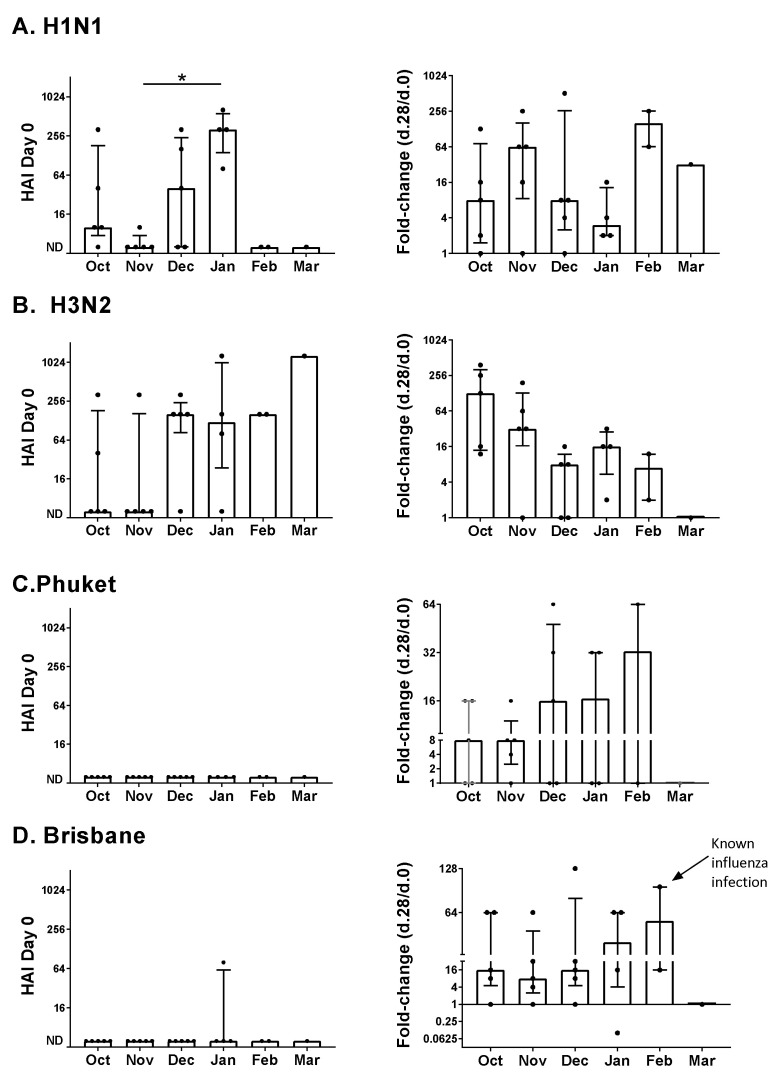
Hemagglutination inhibition (HAI) responses by month of vaccination in a pediatric influenza vaccine study. Participants (*n* = 22) received the influenza virus vaccine during the 2016–2017 season. They were vaccinated in October (*n* = 5), November (*n* = 5), December (*n* = 5), January (*n* = 4), February (*n* = 2), and March (*n* = 1). Antibody titers were scored as the highest dilution that demonstrated HAI when replicates were identical or the average dilution when replicates were not identical. Negative scores (ND = not detected) were assigned a value of 5. Baseline (Day zero) HAI titers (**left**) and fold-changes in titers (**right**, Day 28 compared to Day zero) are shown. Target viruses were (**A**). H1N1 (A/California/7/2009 H1N1). (**B**). H3N2 (A/Switzerland/9715293/2013 H3N2), (**C**). Phuket (B/Phuket/3073/2013), and (**D**). Brisbane (B/Brisbane/60/2008). Data were categorized by the month of vaccination with medians and interquartile ranges. Each symbol (dot) represents a different individual. Significant differences between months were determined by the Kruskal–Wallis test with the Dunn’s multiple comparisons test. Statistical significance is indicated with an asterisk (* *p* < 0.05).

A study was performed to evaluate the effect of inoculation month on the immune response to influenza virus vaccines. Samples were from healthy children between the ages of two and eight years who received the influenza virus vaccine between October and March of 2016–2017. Participants had been part of a placebo group in a clinical study of vitamin supplementation [3]. Participants were grouped based on their month of vaccination (Figure 1).

Baseline serum HAI titers (Day zero, Figure 1, left column) and changes post-vaccination (Day 28 compared to Day zero, Figure 1, right column) are shown for each of the vaccine components. HAI titers toward H1N1 and H3N2 viruses were often detectable at baseline (12/22 and 13/22 positive responses, respectively, at baseline), whereas HAI titers toward Phuket and Brisbane viruses were rarely detectable at baseline (0/22 and 1/22 positive responses, respectively, at baseline). This may reflect the frequent circulation of type A influenza viruses and the likelihood that study participants were exposed prior to study enrollment.

Results in Figure 1 indicated an influence of month on HAI titers. For the H1N1 vaccine component, when baseline titers were compared (Figure 1A), we found that participants enrolled in the month of January exhibited the highest median titers (*p* = 0.039 for the November–January comparison, Kruskal–Wallis with Dunn’s multiple comparisons tests). In contrast, the fold-change median values on Day 28 (Day 28/Day zero) were lowest in January. We further observed that for the H3N2 vaccine component, participants enrolled in the winter months (December, January, and February) exhibited higher median baseline titers than participants enrolled in the fall (October or November, *p* = 0.046, Mann–Whitney test) and that changes in HAI titers post-vaccination (Day 28) were significantly lower for participants enrolled in the winter months compared to the fall (*p* = 0.0072, Mann–Whitney test).

For the Phuket and Brisbane vaccine components, the median changes in HAI titers post-vaccination trended higher in the winter months compared to the fall. There was a known influenza infection of one participant who was vaccinated in February. This individual was diagnosed with influenza shortly after vaccination and exhibited one of the highest responses toward the Brisbane vaccine component (Figure 1D).

### 3.2. 2017–2018 Vaccine Study

We next evaluated an adolescent/adult influenza vaccine study performed during the 2017–2018 influenza virus season. Vaccinations were administered between September and February. There were 271 participants between the ages of 12 and 83 years. We again examined baseline antibody HAI titers and fold-change HAI titers, in this case, comparing Day 21 with Day zero titers.

**Figure 2:**
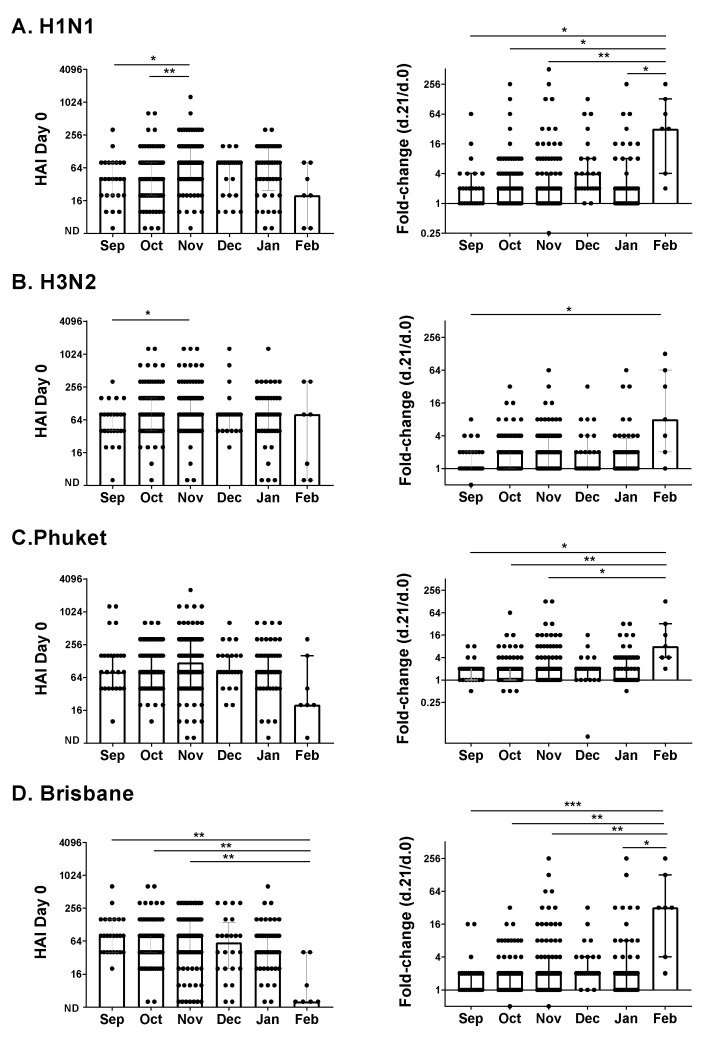
HAI responses by month of vaccination in an adolescent/adult influenza vaccine study. Participants (*n* = 271) received the influenza virus vaccine during the 2017–2018 season. They were vaccinated in September (*n* = 25), October (*n* = 83), November (*n* = 88), December (*n* = 24), January (*n* = 44), and February (*n* = 7). Baseline (Day zero) HAI titers and fold-change values (Day 21 compared to Day zero titers) are shown as symbols (dots) for each volunteer. Negative scores (ND = not detected) were assigned a value of 5. Target viruses were (**A**). H1N1(A/Michigan/45/2015 H1N1), (**B**). H3N2 (A/Hong Kong/4801/2014 H3N2), (**C**). Phuket (B/Phuket/3073/2013), and (**D**). Brisbane (B/Brisbane/60/2008). Data were categorized by the month of vaccination, and analyzed as in Figure 1. Results of statistical analyses are shown by asterisks (* *p* < 0.05; ** *p* < 0.01; *** *p* < 0.005).

Similar to the 2016–2017 study, we found that the month of inoculation influenced HAI titers. As shown in Figure 2, there were several significant differences when HAIs were compared by the month of enrollment using rank-based tests (Kruskal–Wallis with Dunn’s multiple comparisons), both for Day zero values and fold-change (Day 21 compared to Day zero) values. The highest fold-change values were in February. There was also a difference between fall (September, October, and November) and winter (December, January, and February) months; the Brisbane baseline titers were significantly higher in the fall than in the winter by rank (*p* = 0.0045, Mann–Whitney test).

### 3.3. Age and Responses to the Influenza Vaccine

**Figure 3:**
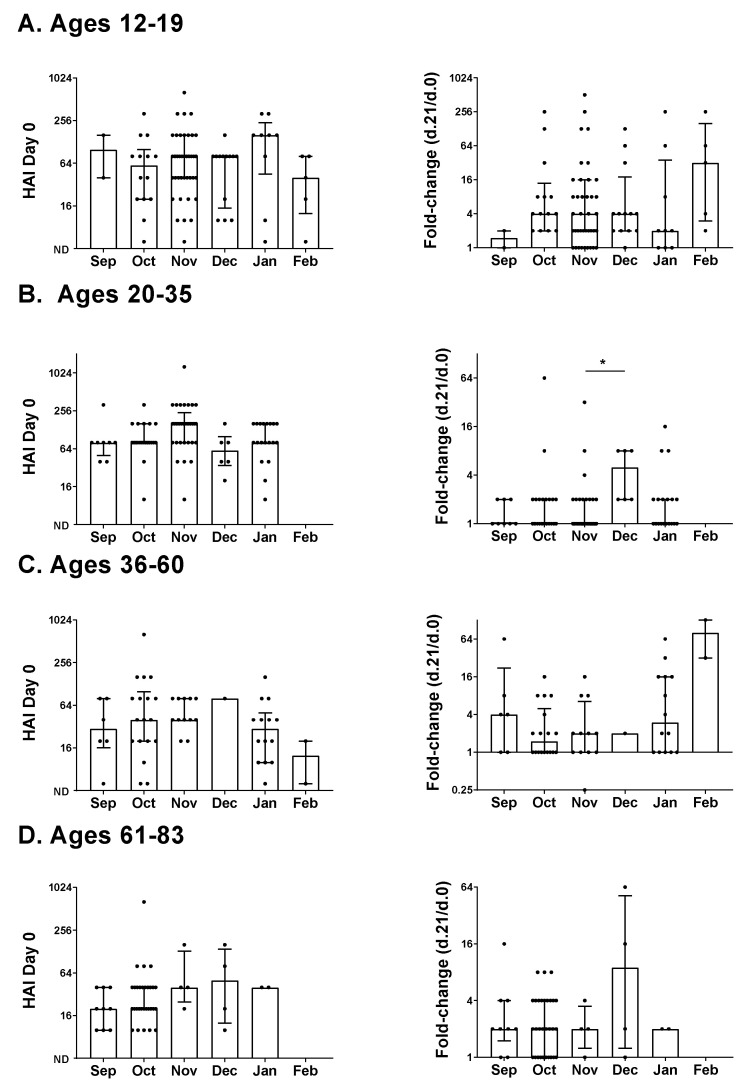
Age and HAI responses by month of vaccination in an adolescent/adult influenza vaccine study. Baseline HAI titers and fold-change results for the 2017–2018 season for participants categorized by age (**A**). 12–19, (**B**). 20–35, (**C**). 36–60, and 9 (**D**). 61–83, inclusive are shown against the H1N1 virus (A/Michigan/45/2015 H1N1). Values (presented as dots for each individual) and statistics are as described in Figure 1. Negative scores (ND = not detected) were assigned a value of 5. Statistical significance is indicated with an asterisk (* *p* < 0.05).

We asked if age contributed to HAI differences in the 2017-2018 adolescent/adult study (Figure 3). To examine age, we focused on the H1N1 vaccine component. Participants were categorized by age: 12–19 (*n* = 82), 20–35 (*n* = 87), 36–60 (*n* = 53), and 61–83 (*n* = 49). The medians for Day zero HAI values with all months combined were highest in the two lower age groups (medians were 80 for ages 12–19, 80 for ages 20–35, 40 for ages 36–60, and 20 for ages 61–83). The median fold-change value was highest in the lowest age group (medians were four for ages 12–19, one for ages 20–35, 2 for ages 36–60, and two for ages 61–83) [4]. For all age groups, when individual months were compared, the highest median fold-change was in the winter.

### 3.4. Circulating Influenza Viruses during the 2016–2017 and 2017–2018 Influenza Seasons

**Figure 4:**
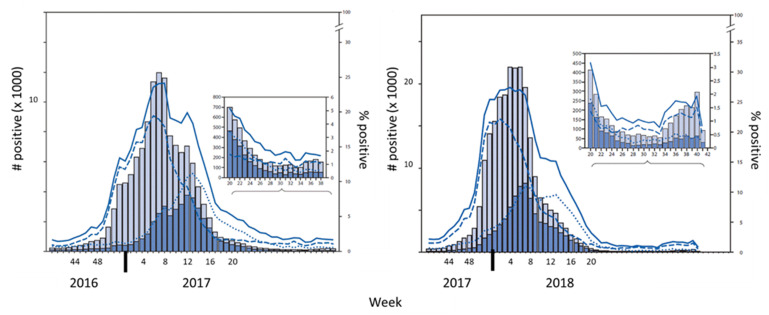
Virus infections during 2016–2017 and 2017–2018 influenza virus seasons. Results are adapted from Centers for Disease Control and Prevention (CDC) reports of influenza virus cases during the 2016–2017 influenza virus season (**left**) [5] and the 2017–2018 influenza virus season (**right**) [6]. Weeks of the year are shown on X axes. Grey bars: numbers of positives for influenza A, blue bars: numbers of positives for influenza B (left *Y*-axes), Solid line: % respiratory samples testing positive, Dashed line: % positive for influenza A, Dotted line: % positive for influenza B (right *Y*-axes).

We next reviewed influenza virus infections reported to the Centers for Disease Control and Prevention (CDC) in the United States during the 2016–2017 and 2017–2018 influenza virus seasons (Figure 4). Infections were scored as positive for influenza by the CDC when influenza antigens and/or nucleic acids were detected in patient samples [5,6].

Influenza-like illnesses began to rise in the fall of 2016 in Tennessee (reported by the Tennessee outpatient influenza-like illness surveillance Network (ILINet) and by the CDC flu surveillance site at Vanderbilt University), including Shelby County where our pediatric clinical study was performed. Type A (H3) virus infections were identified early in the 2016–2017 season, possibly influencing the H3-specific immune responses that we observed pre- and post-vaccination (Figure 1).

For both years, the circulating influenza virus in the United States was highest during the winter months, with peaks toward the end of February (week eight). Based on cumulative results, it is likely that the circulation of influenza viruses contributed to the HAI titers and immune responses in our pediatric and adolescent/adult influenza vaccine studies.

## 4. Discussion

Results reveal significant influences of the month of vaccination on fold-change in HAI antibody titers post-vaccination. Influences were observed both in the 2016–2017 and 2017–2018 studies.

Why did study results change from month to month? A simple explanation is that influenza virus circulation changed between months, that study participants were frequently exposed to virus, and that immune responses to natural virus infections distorted study results. Most notable in the pediatric study was one child who was known to have been naturally infected with influenza virus and who exhibited one of the highest responses toward the Brisbane antigen. It has already been reported that a significant number of individuals are sub-clinically infected with influenza viruses during each flu season [7,8]. If a clinical or subclinical influenza virus infection occurred during or after vaccine dosing (as for the child vaccinated in February during the 2016–2017 pediatric study), it may have amplified antibody titers and lent to a false-positive or exaggerated score. In contrast, if an infection occurred prior to vaccine dosing, it may have amplified baseline titers; these high antibody titers might then have cleared the vaccine so quickly that an immune response toward the vaccine was not observed. We and others have previously found that immune responses toward viral vaccines can be inversely correlated with baseline serum antibody levels [9].

Changes between months in immune responses post-vaccination may also be influenced by non-flu pathogens, particularly if individuals are exposed to cross-reactive antigens or factors that drive polyclonal B cell activation. Additional changes that will influence B cell functions between fall and winter months include changes in sunlight and vitamin levels [10], each of which can alter baseline HAI activities. Further research is now needed to: (i) compare non-flu and flu-specific immune responses based on the month of vaccination, and (ii) examine nutritional and other lifestyle factors that correspond with antibody baseline and fold-change profiles.

A limitation of our research was that we only evaluated two vaccination studies. We note that month-to-month changes in study data were not identical between the 2016–2017 and 2017–2018 studies. This is not surprising, given that circulating viruses and participant characteristics varied between studies. We recommend that in future clinical vaccine studies, independent assessments of vaccination month are performed.

## 5. Conclusions

Results described in this report indicate that the month of vaccination influences the immune responses toward influenza virus vaccines. This may be due in part to the high frequency of clinical and subclinical virus infections that occur during the fall and winter months. This concept has broad significance and will affect the development of most vaccines (including the COVID-19 vaccines that are being developed to combat the current SARS-CoV-2 pandemic [11]). Along with subclinical/clinical infections, there are host lifestyles (e.g., diet, sun exposure) that may change between months and affect immune responses. Attention to the month of inoculation, intercurrent infections, and lifestyle changes are now warranted in future studies to avoid misinterpretation of study data and to expedite the development of successful vaccines in influenza and non-influenza virus fields.

## Data Availability

Data are contained within the article. Additional details are available upon request from the corresponding author.

## Abbreviations

HA	Hemagglutinin
HAI	hemagglutination inhibition
IM	Intramuscular
RBC	red blood cells
